# Effects of Pulsed Electromagnetic Field on Differentiation of HUES-17 Human Embryonic Stem Cell Line

**DOI:** 10.3390/ijms150814180

**Published:** 2014-08-14

**Authors:** Yi-Lin Wu, Shi-Rong Ma, Tao Peng, Zeng-Hui Teng, Xiang-Yan Liang, Guo-Zhen Guo, Hai-Feng Zhang, Kang-Chu Li

**Affiliations:** 1 Department of Radiation Medicine, the Fourth Military Medical University, Xi’an 710032, China; E-Mails: wuyilin710032@hotmail.com (Y.-L.W.); kangchu@fmmu.edu.cn (G.-Z.G.); 2 Experiment Teaching Center, the Fourth Military Medical University, Xi’an 710032, China; E-Mail: liangxiangyan710032@hotmail.com; 3 Department of Pathology and Pathophysiology, the Fourth Military Medical University, Xi’an 710032, China; E-Mail: shirongm@gmail.com; 4 Department of Neurosurgery, the First Hospital of Jilin University, Changchun 130021, China; E-Mail: flwrdng@gmail.com; 5 Department of Pharmaceutics, the Fourth Military Medical University, Xi’an 710032, China; E-Mail: tengzenghui710032@hotmail.com

**Keywords:** PEMF, hESC, differentiation

## Abstract

Electromagnetic fields are considered to potentially affect embryonic development, but the mechanism is still unknown. In this study, human embryonic stem cell (hESC) line HUES-17 was applied to explore the mechanism of exposure on embryonic development to pulsed electromagnetic field (PEMF) for 400 pulses at different electric field intensities and the differentiation of HUES-17 cells was observed after PEMF exposure. The expression of alkaline phosphatase (AP), stage-specific embryonic antigen-3 (SSEA-3), SSEA-4 and the mRNA level and protein level of Oct4, Sox2 and Nanog in HUES-17 cells remained unchanged after PEMF exposure at the electric field intensities of 50, 100, 200 or 400 kV/m. Four hundred pulses PEMF exposure at the electric field intensities of 50, 100, 200 or 400 kV/m did not affect the differentiation of HUES-17 cells. The reason why electromagnetic fields affect embryonic development may be due to other mechanisms rather than affecting the differentiation of embryonic stem cells.

## 1. Introduction

The development of electromagnetic field application has increased the possibility of human exposure to this potentially harmful factor and raised concern as to its possible ill-health effects. Some previous studies reported that electromagnetic field might affect the development of embryo [1,2,3,4,5,6]. But the mechanism is still unclear. Since numerous experiments cannot be conducted in human embryo because of ethical limitations, and a large number of animals are required to evaluate the genotoxicity of electromagnetic fields during embryonic development, as an alternative, use of human embryonic stem cells (hESCs) would be a feasible proposition [7]. The hESCs can differentiate into any cell type found in the human body. In other words, these cells have the ability to become virtually any cell of any of the three germ layers (endoderm, ectoderm, mesoderm). It is for this reason that we are studying the bioeffects of electromagnetic field on hESCs, in order to explore the mechanism of the teratogenic effects of an electromagnetic field and to understand the effects of an electromagnetic field on human heredity and development more directly.

It has been reported that births involving assisted reproductive technology (ART) may have an increased risk of imprinting disorders such as Beckwith–Wiedemann syndrome and Angelman syndrome [8]. Furthermore, in addition to reports of low birth weight and chromosomal anomalies, there is evidence that ART may be associated with increased epigenetic disorders in infants who are conceived using ART, such as *in vitro* fertilization (IVF) and intracytoplasmic sperm injection (ICSI) [9]. One of the reasons for the decrease in success rate as well as an increase in malformations may be due to exposure of the stem cells in early embryonic development to an electromagnetic field during incubation before implantation [10].

So we hypothesized that the effects on embryonic development caused by an electromagnetic field could result from disturbing the proliferation, differentiation or other physiological process of hESCs. The disturbance on expression of regulation related molecules maybe leads to embryogenesis disorder or functional defect. In this study, we focused on the differentiation of hESCs. Therefore, the differentiation of hESC line HUES-17 was observed after pulsed electromagnetic field (PEMF) exposure at different electric field intensities in order to explore the mechanism of electromagnetic field on embryonic development.

## 2. Results and Discussion

### 2.1. Effects of Pulsed Electromagnetic Field (PEMF) on Undifferentiated Markers in HUES-17 Cells

**Figure 1:**
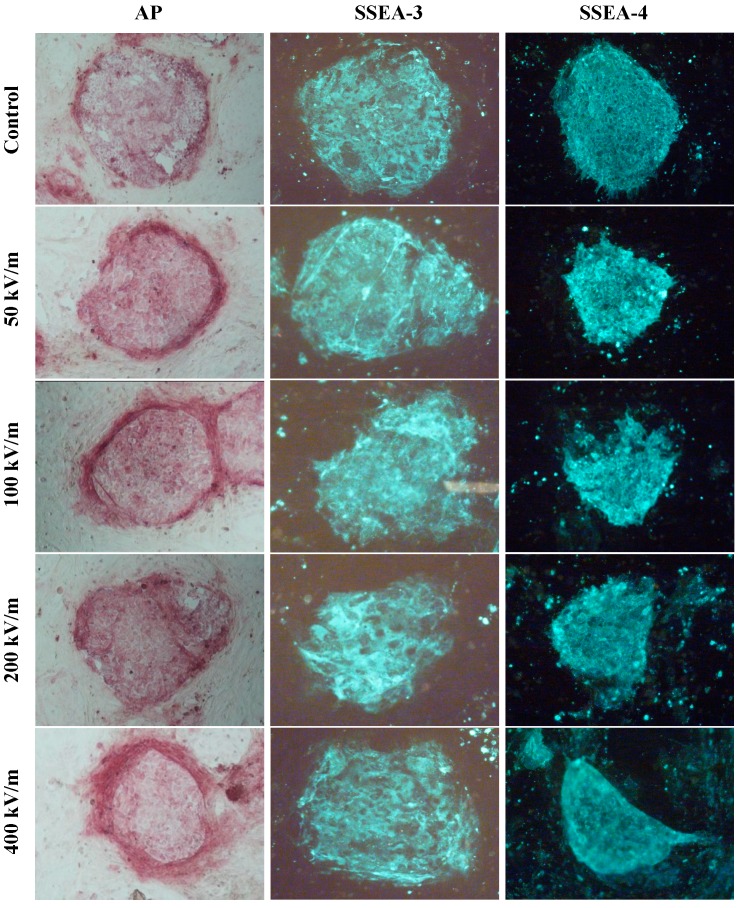
Expression of human embryonic stem cell (hESC) undifferentiated markers in HUES-17 cells after exposed to pulsed electromagnetic field (PEMF) at different electric field intensities. The expression of alkaline phosphatase (AP), stage-specific embryonic antigen-3 (SSEA-3) and SSEA-4 in HUES-17 cells was detected by AP staining and immunofluorescent staining after the cells were exposed to 400 pulses PEMF at electric field intensities of 50, 100, 200, 400 kV/m or sham-exposed (control group). Abbreviations: AP, alkaline phosphatase; SSEA, stage-specific embryonic antigen; PEMF, pulsed electromagnetic field.

To detect the effects of PEMF at different electric field intensities on undifferentiated markers in hESCs, the expression of alkaline phosphatase (AP), SSEA3 and SSEA4 in HUES-17 cells was detected after the cells were exposed to 400 pulses PEMF at electric field intensities of 50, 100, 200, 400 or 0 kV/m (control group). HUES-17 cells in each group expressed undifferentiated hESC markers AP, SSEA3 and SSEA4. But there was no significant difference between PEMF exposed group (50, 100, 200, 400 kV/m) and the corresponding control group. Also, no significant difference was found in expression of AP, SSEA3 and SSEA4 in HUES-17 cells among PEMF exposed groups (Figure 1).

### 2.2. Effects of PEMF on mRNA Level of Oct4, Sox2 and Nanog in HUES-17 Cells

**Figure 2:**
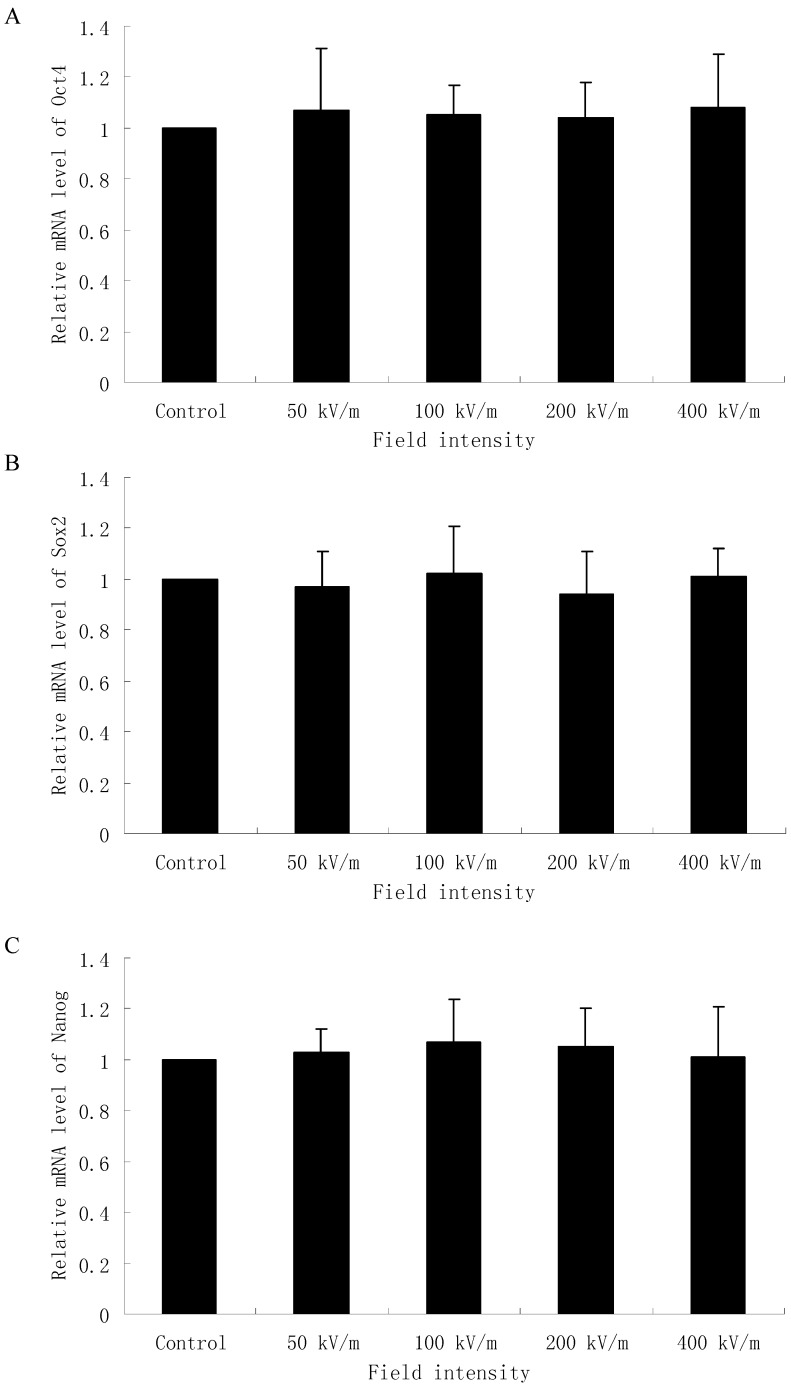
Real-time polymerase chain reaction analysis of *Oct4* (**A**), *Sox2* (**B**) and *Nanog* (**C**) gene expression in HUES-17 human embryonic stem cells (hESCs) after exposed to PEMF at different electric field intensities (control group, 50, 100, 200, 400 kV/m). The expression value of each gene was normalized by an internal control gene glyceraldehyde-3-phosphate dehydrogenase (*GAPDH*). The expression level of each gene in the control group is arbitrarily defined as one unit. Results were obtained from three independent experiments and expressed as mean *±* SD (*n* = 3 in each experiment).

Since *Oct4*, *Sox2* and *Nanog* are three key transcription factors which play an important role in maintaining self-renewal, or pluripotency, of undifferentiated embryonic stem cells, we examined the mRNA level of *Oct4*, *Sox2* and *Nanog* in HUES-17 cells exposed to PEMF at different field intensities. As shown in Figure 2, 400 pulses PEMF exposure at 50, 100, 200, 400 kV/m cannot significantly affect the mRNA level of *Oct4*, *Sox2* and *Nanog* in HUES-17 cells compared with the control group (*p* > 0.05).

### 2.3. Effects of PEMF on Protein Level of Oct4, Sox2 and Nanog in HUES-17 Cells

**Figure 3:**
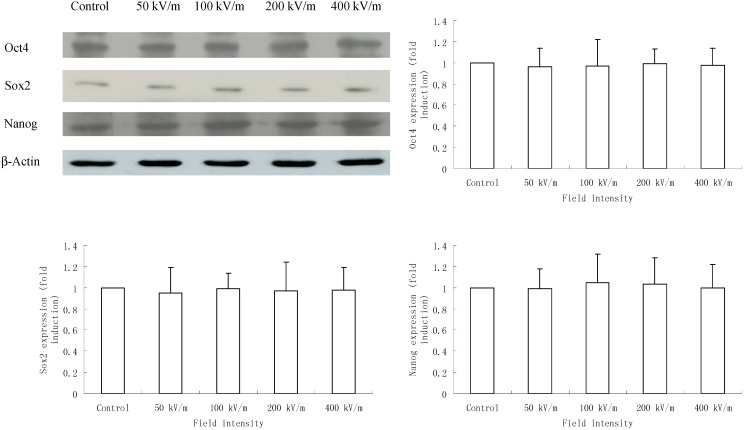
Western blotting analysis of Oct4, Sox2 and Nanog expression in HUES-17 human embryonic stem cells (hESCs) after exposed to PEMF at different electric field intensities (control group, 50, 100, 200, 400 kV/m). β-Actin was used as an internal control. Protein level of each gene was quantitated by densitometry and plotted as fold induction. Results were obtained from three independent experiments and expressed as mean *±* SD (*n* = 3 in each experiment).

We further detected the protein level of Oct4, Sox2 and Nanog in HUES-17 cells exposed to PEMF at different field intensities and found that in accordance with the real-time PCR results, after 400 pulses PEMF exposure, HUES-17 cells in all groups (control group and 50, 100, 200, 400 kV/m exposed groups) expressed Oct4, Sox2 and Nanog protein. However, the protein level of Oct4, Sox2 and Nanog in HUES-17 cells in PEMF exposed group (50, 100, 200, 400 kV/m) was not significantly changed compared with that in the corresponding control group (*p* > 0.05) (Figure 3).

### 2.4. Discussion

In recent years, more and more concern has been shown for the effects of electromagnetic fields on embryonic stem cells because these cells have extensive proliferative potential and display a capacity for multilineage differentiation, therefore offer an important area of study. For instance, Czyz *et al.* [11,12] studied the effect of electromagnetic fields *in vitro* on wild type ES cells and ES cells deficient for the tumor suppressor gene *TP53*. The applied electromagnetic fields induced a significant up-regulation of mRNA levels of the heat shock protein hsp70, along with a low and transient increase of c-jun, c-myc, and p21 levels in p53 deficient embryonic stem cells *in vitro*, but not in wild type cells.

There is some evidence to support the contention that electromagnetic fields might affect the differentiation process [13,14,15,16,17,18,19], However, as for the effects of electromagnetic fields on the differentiation of embryonic stem cells, only a few data are available. Serena *et al*. [20] found that electromagnetic field stimulation plays a role in cardiac differentiation of hESCs. Ventura *et al.* [21] showed that exposure of mouse embryonic stem cells to ELF-electromagnetic fields triggered the expression of cardiac lineage-promoting genes. Sauer *et al.* also noted that exposure of mouse embryonic stem cells to electromagnetic fields was found to promote cardiomyogenic differentiation [22]. The previous reports showed that different electromagnetic fields might have different biological effects. Recently Yang *et al.* in our lab reported that PEMF exposure (400 kV/m with 400 pulses) increased the rates of polydactyly fetuses of mice [6]. Therefore, to investigate the underlying mechanism related to embryonic stem cell differentiation, for the current we chose the same PEMF with high electric field intensity for the stimulation to study the effects of PEMF on embryonic development.

It is well known that AP, SSEA3 and SSEA4 are undifferentiated hESC markers. The expression of these molecules is considered as maintaining the undifferentiation characteristics of hESCs. So far, there is no report available regarding the effects of PEMF on expression of AP, SSEA3 and SSEA4 in hESCs. Under our experimental conditions, we could not find any significant changes in undifferentiated hESC markers AP, SSEA3 and SSEA4 after the HUES-17 cells were exposed to 400 pulses PEMF at electric field intensities of 50, 100, 200, 400 kV/m which indicated that differentiation of hESCs was not affected by electromagnetic field at the different electric field intensities we used.

Oct4 is critically involved in the self-renewal of undifferentiated embryonic stem cells [23]. Oct4 expression must be closely regulated; too much or too little will cause differentiation of the cells [24]. Sox2 is a transcription factor that is essential for maintaining self-renewal, or pluripotency, of undifferentiated embryonic stem cells. Sox2 also has a critical role in maintenance of embryonic stem cells [25]. Nanog is another transcription factor critically involved with self-renewal of undifferentiated embryonic stem cells. Nanog is thought to be a key factor in maintaining pluripotency and to function in concert with other factors such as Oct4 and Sox2 to establish embryonic stem cell identity.

In the present study, we also detected the expression of these three critical transcription factors, Oct4, Sox2 and Nanog, in HUES-17 cells after 400 pulses PEMF exposure at electric field intensities of 50, 100, 200 or 400 kV/m in order to explore whether the PEMF at different electric field intensities could affect the expression of these three critical transcription factors related to undifferentiation of hESCs. Our data showed that neither the mRNA level nor the protein level of Oct4, Sox2 and Nanog in HUES-17 cells were altered by our applied PEMF. These three transcription factors could not be affected by PEMF at different electric field intensities which was consistent with our previous results and might be the possible reason for the lack of effects on undifferentiated hESC markers AP, SSEA3 and SSEA4 expression in HUES-17 cells after PEMF exposure.

## 3. Experimental Section

### 3.1. Human Embryonic Stem Cell (hESC) Culture

The hESC line HUES-17 was kindly provided by Douglas Melton, Harvard University [26]. All hESC experiments were conducted in accordance with the guidelines for research on hESCs, jointly issued by the Ministry of Science and Technology and the Ministry of Health of China [27], and approved by the ethical committee of Fourth Military Medical University (National Natural Science Foundation of China No. 30800928, 09/01/2009). HUES-17 cells were maintained on feeders in hESC medium, which contained 80% Dulbecco’s modified Eagle’s medium/Ham’s F-12 medium (F12), 20% knock-out serum replacement, 1 mM l-glutamine, 0.1 mM β-mercaptoethanol, 1% nonessential amino acids, and 4 ng/mL human basic FGF (GIBCO, Grand Island, NY, USA). HUES-17 cells were passaged approximately once a week by incubation in 1 mg/mL collagenase IV (GIBCO, Grand Island, NY, USA) for 30 min at 37 °C.

### 3.2. PEMF Exposure

The PEMF exposure device was described by Li *et al*. [28]. Briefly, the PEMF (a pulsed electromagnetic wave at a repetition rate of 0.5 Hz, pulse-width 350 ns, peak-intensity 400 kV/m) was generated by a spark gap pulse generator. A tapered parallel plate Gigahertz Transverse Electromagnetic cell (GTEM cell) with a flared rectangular coaxial transmission line was used to expose the HUES-17 cells. The PEMF generator and the GTEM-cell were both devised by the Department of Mechanical Engineering, Southeast University (Nanjing, China). To study the effects of PEMF at different electric field intensities on differentiation of human embryonic stem cells, HUES-17 cells in culture were exposed to PEMF for 400 pulses (about 13 min) at the electric field intensities of 50, 100, 200, 400 kV/m or were sham-exposed (control group). The temperature measurements were done with a thermometer immediately before and after PEMF exposure. The exposure produced a rise in the culture medium temperature less than 0.1 °C.

### 3.3. Alkaline Phosphatase (AP) Staining

AP staining was performed with ES Cell Characterization Kit (Millipore, Billerica, MA, USA) according to the manufacturer’s instructions. Briefly, 24 h after the PEMF exposure, the HUES-17 cells were fixed with 4% paraformaldehyde in PBS for 2 min at room temperature. Subsequently, HUES-17 cells were rinsed with TBST (20 mM Tris–HCl, pH 7.4, 150 mM NaCl, 0.05% Tween-20) three times and then stained with AP solution in the dark at room temperature for 15 min. The stained cells were observed under an inverted microscope after three rinses with TBST.

### 3.4. Immunofluorescence Staining for Specific Markers

Immunofluorescence staining was carried out similarly as described [29]. Briefly, HUES-17 cell colonies were rinsed with TBST (20 mM Tris–HCl, pH 7.4, 150 mM NaCl, 0.05% Tween-20) 24 h after the PEMF exposure and then fixed with 4% paraformaldehyde in PBS for 20 min at room temperature. After washing with TBST for 15 min, the cells were permeablized with 0.1% Triton X-100 in PBS for 10 min at room temperature, followed by three rinses with TBST prior to incubation with blocking solution (4% normal goat serum/PBS) for 30 min at room temperature. The putative HUES-17 cells were first incubated with primary antibodies: Anti- stage-specific embryonic antigen-3 (SSEA-3), anti-SSEA-4 (Chemicon, Billerica, MA, USA). All antibodies were diluted in blocking solution (1:25) and incubated with samples for 60 min at room temperature. After incubation with the primary antibody, the HUES-17 cells were washed again with TBST three times and then incubated with the secondary antibodies (FITC-conjugated goat anti-mouse IgG, 1:200, Sigma, St. Louis, MO, USA) for 60 min at room temperature. Finally, after three rinses with TBST the fluorescence images were visualized with a fluorescence microscope.

### 3.5. RNA Extraction and Real-Time Reverse Transcription-Polymerase Chain Reaction

Twenty four hours after the PEMF exposure, the cells were rinsed twice with ice-cold PBS and scraped into TRIZOL (Invitrogen, Carlsbad, CA, USA). Total RNA of HUES-17 cells was extracted according to the manufacturer’s instructions. cDNA was synthesized using the PrimeScript™ RT Master Mix First Strand cDNA Synthesis Kit (TaKaRa, Shiga, Japan). Real-time PCR was performed using a SYBR^®^ Premix Ex Taq™ II PCR Kit (TaKaRa, Shiga, Japan). Primers of the selected genes for PCR reactions were synthesized (Sunbiotech Co., Beijing, China) as in Table 1 [30,31]. The expression value of each gene was normalized to the amount of glyceraldehyde-3-phosphate dehydrogenase (GAPDH) cDNA to calculate a relative amount of RNA present in each sample. The expression level of each gene in a single sample was arbitrarily defined as one unit. The normalized expression values for all control and treated samples were averaged, and an average-fold change was determined. Analysis of variance was conducted between the normalized relative expression values for control and treated samples to determine statistical significance. At least three independent experiments from cell culture to real-time PCR were conducted, and each real-time PCR was performed at least three times.

**Table 1:** Primers used for real-time polymerase chain reaction.

Gene Name	Primer
*Oct4*	Forward: AGAAGGATGTGGTCCGAGTGTG
	Reverse: CCACCCTTTGTGTTCCCAATTCC
*Sox2*	Forward: CCCCCGGCGGCAATAGCA
	Reverse: TCGGCGCCGGGGAGATACAT
*Nanog*	Forward: TGAACCTCAGCTACAAACAGGTG
	Reverse: AACTGCATGCAGGACTGCAGAG
*GAPDH*	Forward: AGGTCGGAGTCAACGGATTTGG
	Reverse: AGGCTGTTGTCATACTTCTCATGG

### 3.6. Western Blotting

Twenty four hours after the PEMF exposure, the cells were rinsed twice with ice-cold PBS and scraped into lysis buffer [20 mM Tris–HCl, pH 8.0, 2% Nonident P-40, 150 mM NaCl, 5 mM MgCl_2_, 5 mM ethylene diamine tetraacetie acid (EDTA), 2 mM NaN_3_, and 1 mM phenylmethylsulfonyl fluoride (PMSF)]. After centrifugation at 12,600× *g* for 10 min at 4 °C, the supernatants (20 μg protein/sample) were separated on sodium dodecyl sulfate (SDS)-10% polyacrylamide gel. After electrophoresis, the separated proteins were blotted onto a nitrocellulose membrane (Osmonics, Minnetonka, MN, USA) using a semi-dry electroblotter, and the membrane was blocked at room temperature for 2 h in 5% non-fat milk in Tris buffered saline with Tween 20 (TBST) (10 mM Tris–HCl, pH 7.5, 150 mM NaCl, and 0.05% Tween 20). Subsequently, the membrane was incubated at 4 °C overnight with the following primary antibodies: anti-Oct4 (Santa Cruz Biotechnology Inc., Santa Cruz, CA, USA), anti-Sox2 (Millipore, Billerica, MA, USA), anti-Nanog (R&D Systems, Minneapolis, MN, USA) and anti-β-actin (Abcam, Cambridge, MA, USA). The bound antibody was detected with the horseradish peroxidase (HRP)-conjugated secondary antibody (HRP-conjugated goat anti-rabbit antibody and HRP-conjugated rabbit anti-goat antibody, Zhongshan Goldenbridge Biotechnology Co., Beijing, China). The desired proteins were visualized by use of the ECL Western blotting detection system (Pierce, Rockford, IL, USA) according to the manufacturer’s instructions. The protein bands were quantified using QuantityOne software (Bio-Rad, Hercules, CA, USA) and the protein expression levels were normalized to the expression of a housekeeping protein, β-actin. At least three independent experiments from cell culture to Western Blot were conducted, and each Western Blot was performed at least three times.

### 3.7. Statistical Analysis

The data are expressed as mean ± standard deviation (SD). Data were analyzed by Student’s *t* test. *p* < 0.05 was considered statistically significant.

## 4. Conclusions

In conclusion, under our experimental conditions PEMF exposure at different electric field intensities did not affect the differentiation of hESCs. Further study focusing on other processes rather than hESC differentiation is needed to explore the mechanism of the teratogenic effects of PEMF.
